# Plastic Roads in Asia: Current Implementations and Should It Be Considered?

**DOI:** 10.3390/ma16165515

**Published:** 2023-08-08

**Authors:** Saipol Bari Abd Karim, Syuhada Norman, Suhana Koting, Khanom Simarani, Siaw-Chuing Loo, Faizul Azli Mohd Rahim, Mohd Rasdan Ibrahim, Nur Izzi Md Yusoff, Abdul Halim Nagor Mohamed

**Affiliations:** 1 Department of Quantity Surveying, Faculty of Built Environment, Universiti Malaya, Kuala Lumpur 50603, Malaysia; siawchuing@um.edu.my (S.-C.L.); azli@um.edu.my (F.A.M.R.); 2 Institute of Biological Sciences, Faculty of Science, Universiti Malaya, Kuala Lumpur 50603, Malaysia; s2000023@siswa.um.edu.my (S.N.); hanom_ss@um.edu.my (K.S.); 3 Center for Transportation Research (CTR), Department of Civil Engineering, Faculty of Engineering, Universiti Malaya, Kuala Lumpur 50603, Malaysia; rasdan@um.edu.my (M.R.I.); 17130030@siswa.um.edu.my (A.H.N.M.); 4 Department of Civil Engineering, Faculty of Engineering and Built Environment, Universiti Kebangsaan Malaysia, Bangi 43600, Selangor, Malaysia; izzi@ukm.edu.my

**Keywords:** plastic roads, Asia, environmental risk, health risk, waste management

## Abstract

The rapid economic and industrial growth experienced in the Asian region has significantly increased waste production, particularly single-use plastic. This surge in waste poses a significant challenge for these countries’ municipal solid waste management systems. Consequently, there is a pressing need for progressive and effective solutions to address the plastic waste issue. One promising initiative involves utilizing used plastic to produce components for asphalt pavement. The concept of plastic road technology has gained traction in Asia, with 32 countries displaying varying levels of interest, ranging from small-scale laboratory experiments to large-scale construction projects. However, as a relatively new technology, plastic road implementation requires continuous and comprehensive environmental and health risk assessments to ascertain its viability as a reliable green technology. This review paper presents the current findings and potential implementation of plastic-modified asphalt in Asian countries, with particular attention given to its environmental and human health impacts. While plastic asphalt roads hold promise in waste reduction, improved asphalt properties, and cost savings, it is imperative to thoroughly consider the environmental and health impacts, quality control measures, recycling limitations, and long-term performance of this road construction material. Further research and evaluation are needed to fully understand the viability and sustainability of plastic asphalt roads. This will enable a comprehensive assessment of its potential benefits and drawbacks, aiding in developing robust guidelines and standards for its implementation. By addressing these considerations, it will be possible to optimize the utilization of plastic waste in road construction and contribute to a greener and more sustainable future.

## 1. Introduction

Over the past 50 years, rapid economic growth in Asia, especially from the manufacturing, fisheries, and tourism sectors, has led to higher incomes, poverty reduction, and a rapidly expanding middle class [1]. However, the increased population growth rate, industrialization, and urbanization strongly correlate to Asia’s plastic intensity and solid waste generation [2]. To manage plastic waste and reduce its adverse impact on the environment, several countries have entered a new phase in applying environmentally sustainable development by implementing green road technology from plastic-modified asphalt [3].

Thermoplastics, commonly known as plastics, are human-made polymers mostly derived from petroleum and natural gas, with a small portion of production from plants such as corn and sugarcane [4]. The first synthetic plastic, Bakelite, was invented by Leo Hendrik Baekeland in 1907 and developed for the industry in the 1920s. During and after World War II, plastic production has grown rapidly due to the scarcity of natural materials [5].

Plastic is one of the most utilized materials in the world, mainly in the textile, automotive, manufacturing, and packaging industries, due to its durability, lightweight, versatility, and relatively low cost of production [6]. The considerable societal benefits of plastics for health, safety, energy saving, and material conservation account for its popularity as a material [7]. Additionally, during the COVID-19 pandemic, the usage of single-use medical plastic products such as disposable syringes, personal protective equipment, masks, gloves, and medical test kits was crucial to reducing the spread of SARS-CoV-2 [8].

Global production of plastic products increased almost 200-fold from 2 million metric tons (Mt) in 1950 to 380 Mt in 2015 [9], which is equivalent to the mass of two-thirds of the world population [10]. Nevertheless, the global demand and production of plastics show a different trend between the European and Asian regions. In European countries, plastic production decreased by 10.3% between 2018 and 2020 [11]. The enforcement of sound policies among plastic manufacturers and improved awareness among European citizens contributed to the reduction of plastic production [12]. On the other hand, Asian countries are well-known as the leading global manufacturer and traders of plastic waste [13]. It was estimated that the total Asian production quantity of major plastic resins in 2017 and 2018 was 82 Mt. China was the largest manufacturer (44.79 Mt), followed by India and Korea with 14.17 and 13.68 Mt, respectively [14].

The projected increase in global plastic usage will lead to a concomitant rise in post-consumer plastic waste. In a business-as-usual scenario, 155 to 265 Mt of mismanaged plastic waste is expected to be produced by 2060 [15]. In the Asian continent, the future mismanaged plastic waste issue will continue to increase due to high production volumes of plastics, insufficiently developed domestic waste management, and lacking recycling systems [16]. As shown in Table 1, the majority of Asian countries showed an increased production of plastic waste from 2016 to 2018 [14].

However, Bangladesh took a proactive step as it became the first country in the world to ban thinner plastic bags in 2002 [17]. This was followed by China, which passed the National Sword policy in 2017 to permanently ban the import of non-industrial global plastic waste [18]. The enforcement of China’s National Sword policy led to the redistribution of the plastic waste trade load to Southeast Asian countries [19].

After more than 100 years of its first invention and usage, plastic eradication has unwittingly become one of the major challenges in our postmodern society [20]. Most single-use plastics, such as in the form of cigarette butts, plastic drinking bottles, plastic bottle caps, food wrappers, plastic grocery bags, plastic lids, straws and stirrers, other types of plastic bags, and take-away foam containers are ubiquitous in the environment [21]. After being used once, most single-use plastics are disposed of in landfills or incinerated, causing the loss of valuable resources. Leachate from landfill areas plays a prominent source of microplastic pollution [22]. Later on, this accumulates and pollutes the water, soil, and air [23].

On top of the environmental pollution problem, the management cost of plastic waste has increased drastically in recent years due to the rise in collection, transportation, and land prices [24,25]. Insufficient infrastructure for collection, reuse, and recycling and high reliance on landfill systems in developing countries emphasize the importance of reusing existing and future plastic products [26]. One of the proposed methods of reusing plastics is in construction materials, for example in bitumen to produce asphalt roads [27].

The development of a country is reliant on its transport system. Despite differences in development patterns across the region, those countries with high transport capacities and efficiency are categorized as the most successful in achieving sustainable development [28]. Integrating economic, social, and environmental pillars into the transport connectivity agenda of Sustainable Development Goals (SDGs) can be achieved by optimizing the needs of transporting goods and passengers; minimizing the consumption of energy, land, and other resources; generating low emissions of greenhouse gases, ozone-depleting substances, and other pollutants; and reducing the adverse social impacts arising from transport operations for both urban and rural communities [29].

By the end of the first quarter of 2022, there were approximately 1.45 billion road vehicles in the world, and about a third of all vehicles are in the Asia–Pacific region [30]. Additionally, 25 million kilometers (km) of roadways will be constructed in many Asian countries by 2050. This translates to an $8.4 trillion investment for transportation projected between 2016 and 2030 [31]. Thus, it highlights the importance of roadways as the main means of terrestrial transport in Asia [32]. The Asian Highway (AH) Network and The Belt and Road Initiative (BRI) are two examples of global infrastructure development strategies that are taking place in Asian countries.

The AH Network (also known as the Great Asian Highway) is a project by the Asian Land Transport Infrastructure Development supported by the United Nations Economic and Social Commission for Asia and the Pacific (ESCAP) to improve transport facilities throughout the 32 participating countries [33]. It was initiated in 1959 with fluctuating progress due to political and economic issues [34]. To date, the total length of the AH Network is 142,774.51 km, with about 70% of the roads categorized as Class II and higher [33,35]. On the other hand, the BRI is a cross-regional economic cooperation framework connecting the China and Asian, African, and European continents, with Central Asia acting as China’s major neighboring diplomacy region [36]. With an estimated cost of more than $4 trillion, The BRI will connect almost half of the global population via land and marine routes [37].

**Figure 1:**
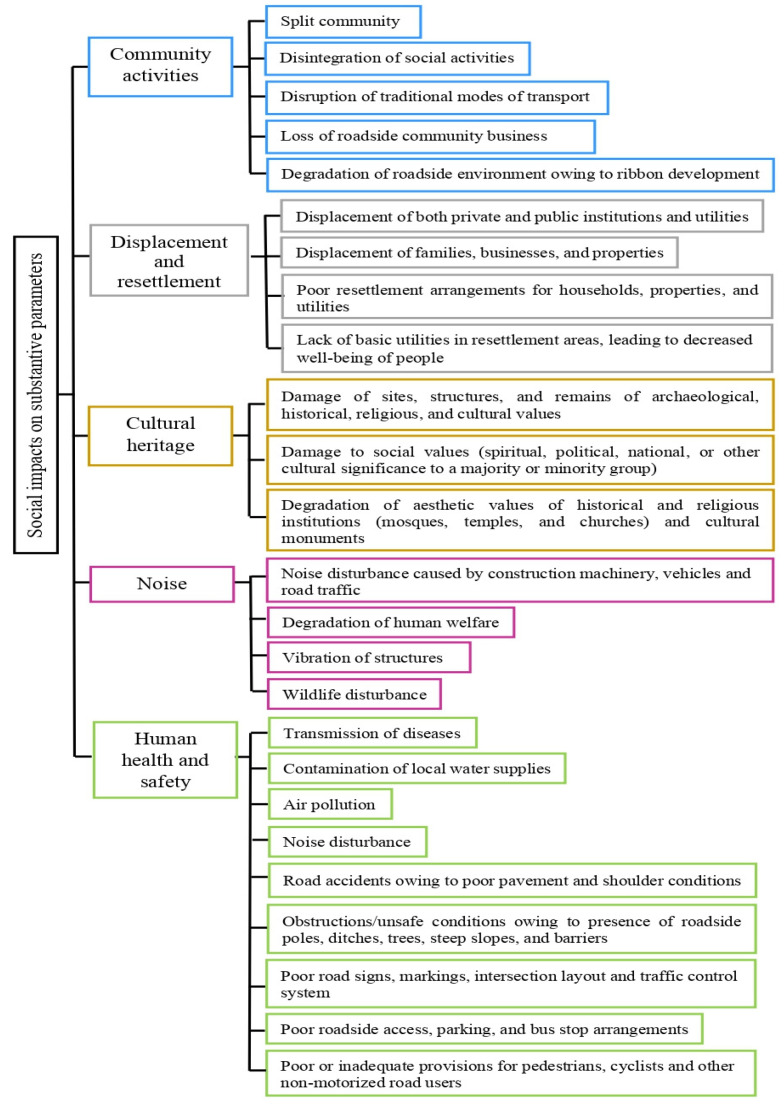
Various negative impacts on the adjacent community may be caused by road development projects [43].

The economic and geopolitical implications of transport systems have been discussed in previous studies [38,39]. The impacts of road and highway development on society, as shown in Figure 1, have also been highlighted in many research papers [40,41,42]. Thus, these issues will not be elaborated in this review paper.

From the public health and safety perspective, human mobilities faced profound changes, especially during the COVID-19 pandemic. Due to travel restrictions as one of the mitigation measures, there were unprecedented falls in aviation, long-distance rail travel, and public transport users. At the same time, there was an increase in land road usage by private vehicle drivers, motorcyclists, cyclists, and pedestrians [44]. Moreover, in low- and middle-income countries, there was significant death among children and adults aged 5 to 29 years caused by road traffic fatalities [45]. Thus, key partners in global road safety have worked together to tackle this public health issue through several road safety performance indicators including financial impact, institutional framework, infrastructure and mobility, legislation and policy, vehicular road users, and trauma management [46].

**Figure 2:**
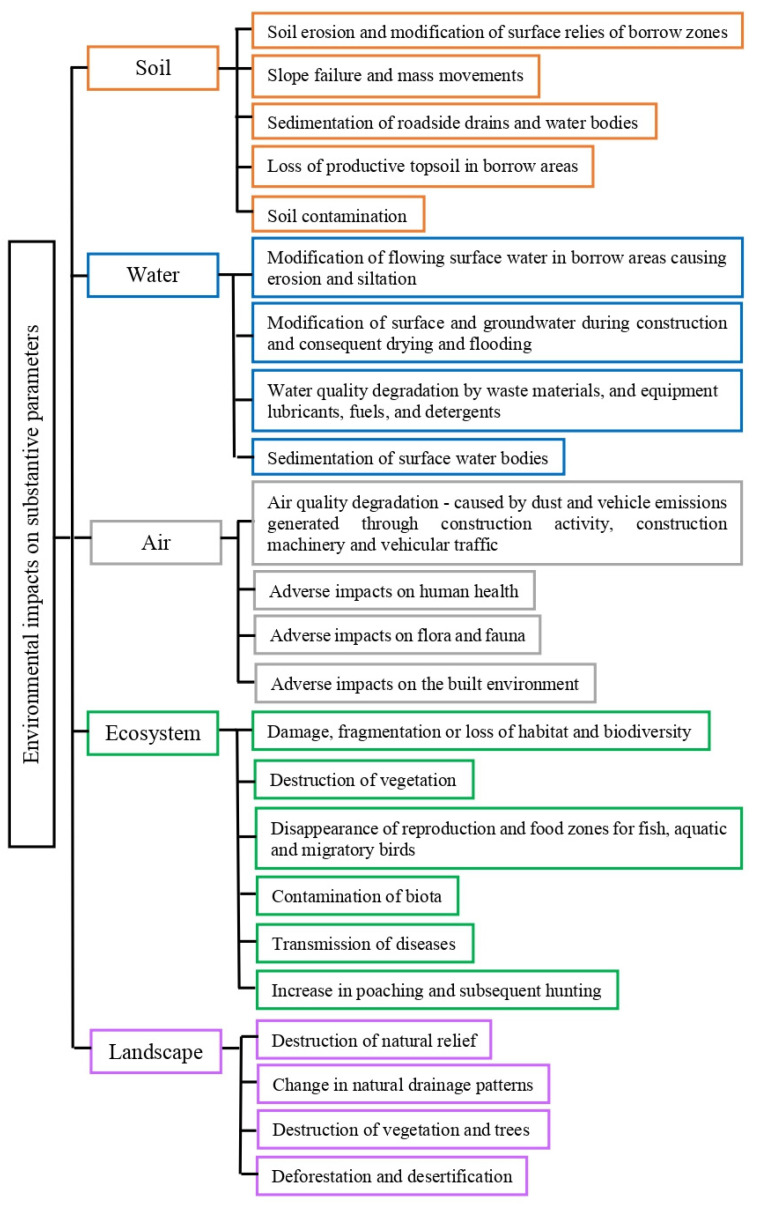
Various negative environmental impacts that may be caused by road development projects [43].

Moreover, the environmental impacts of road development activities must also be evaluated. Poorly planned infrastructures potentially expose disastrous consequences for environmental practices in the coming decades, especially in Asia [47]. For example, roads hinder animal movement, cause direct mortality from vehicle collisions, and reduce their gene flow [48,49]. Traffic disturbances, such as noise, lights, pollution, and traffic motions, lower the quality of nearby habitats [50]. Roads also increase access to previously remote locations, facilitate human settlement growth and exploitation of natural resources, and exacerbate illegal poaching [51,52]. Figure 2 shows major substantive issues of the adverse environmental impacts caused by road development projects.

Environmental evaluation for the road development process is equally crucial as its technical and structural design [53]. The green rating system is an assessment tool to determine the green credential and level of environmental sustainability of a road project [54]. The construction techniques of green roads include the following aspects: the use of recycled materials, ecosystem management, energy reduction, increasing the water quality of stormwater runoff, and maximizing overall societal benefits [55].

Nevertheless, in many developing countries, there are inadequacies of environmental impact assessments, the only safeguard against potential environmental risks and liabilities of road infrastructure projects [56]. Several different types of environmental impact indicators, such as the greenhouse gas footprint, eutrophication potential, acidification potential, human health particulate, ozone depletion, and smog, can be associated with different phases of the construction projects (including manufacturing, transportation, construction, maintenance, operational, recycling, and deconstruction phase) [57]. Hence, this paper will discuss the current implementation of plastic road technology in Asian countries as one of the green initiatives for the road construction industry, emphasizing the environmental and human health impacts and its future potential.

## 2. Research Rationale and Methodology

This review paper will summarize the findings and provide insights into the potential of plastic-modified asphalt technology in Asian countries. It will identify the key advantages, challenges, and research gaps, and offer recommendations for future endeavors to enhance the adoption and sustainability of plastic-modified asphalt technology in a wide range of Asian countries, including but not limited to India, China, Japan, South Korea, Thailand, and Malaysia, to provide a comprehensive regional perspective.

By conducting a comprehensive review of the plastic-modified asphalt technology in Asian countries, this paper aims to contribute to the knowledge base and facilitate informed decision-making for policymakers, researchers, and industry stakeholders in their efforts toward sustainable infrastructure development and plastic waste management.

### 2.1. The Planning Phases

Firstly, the authors planned to review the current implementation of plastic-modified asphalt technology in Asian countries and its possible impacts on the environment and human health. This was followed by structuring the general outline of different sections of the review paper. Based on the outline, the search strategy, sources of literature, and inclusion and exclusion criteria for the literature search were specified.

### 2.2. Data Source and Search Strategy

Desk research using electronic databases, mainly PubMed, Scopus, and Google Scholar were conducted, using a wide range of search terms and keywords or a combination of keywords on the two central concepts, plastic-modified asphalt in Asia and risk, to retrieve the published articles. The keywords included plastic-modified asphalt, plastic roads, plastic asphalt, plastic bitumen, Asia, all Asian countries (i.e., Bangladesh, Saudi Arabia, and China), human health, environmental risk, and life cycle assessment. Second, bibliographic references of these retrieved articles were used to acquire additional relevant papers. Third, the Google search engine was used to attain published discussion papers, reports, and media news. Finally, as a fourth step, several websites were searched to extract and collect published literature.

### 2.3. Inclusion and Exclusion Criteria

The peer-reviewed journal articles, discussion papers, reports, and media news related to the implementation or research on plastic-modified asphalt in different Asian countries and its impacts were included. No exclusions were made based on the article types, methodology of the study, outcome reported, study period, scope of the study, and study setting. Literature was excluded if the language of publication was not found in English.

### 2.4. Data Extraction and Synthesis

Based on the primary outline of the study, the findings were critically summarized, and data were extracted, combined, and synthesized using a narrative synthesis.

## 3. Plastic Roads Construction in Asian Countries

To date, almost 90% of the roads in the world comprise asphalt mixtures combining aggregates, bitumen binder, and filler produced at different temperatures [58]. In conventional asphalt construction, most energy consumption is required to extract, produce, and refine the paving materials [59]. The estimate of total energy consumption during roadway construction is between 3 to 7 terajoules per lane mile [60]. Additionally, conventional asphalt demands more maintenance services to reduce its main problems such as permanent deformation, fatigue cracking, and thermal susceptibility [61]. It also results in abiotic depletion, global warming, and human toxicity risks because of the emulsifying agent component used during the chemical treatment process [62]. Therefore, there is an urgent need for the implementation of sustainable pavements as part of the global effort [63].

The concept of plastic roads involves utilizing waste plastics, such as plastic bags, bottles, and packaging materials, as a component in road construction. Instead of discarding these plastics into landfills or oceans, they are repurposed and integrated into the asphalt mixture used for road paving. The plastic waste is processed and mixed with bitumen, aggregates, and other materials to create plastic-modified asphalt.

Recently, great strides have been made in improving road sustainability, specifically in producing sustainable asphalt pavements [64]. Certain waste products and by-products, such as plastics, marble quarry, building demolition waste, ground tire rubber, cooking oil, palm oil fuel ash, coconut, sisal, cellulose and polyester fiber, starch, waste glass, waste brick, waste ceramic, waste fly ash, and cigarette butts, are being used or intensively developed as aggregates and asphalt binders [65].

### 3.1. Expansion of Plastic Road Idea in Asia

**Figure 3:**
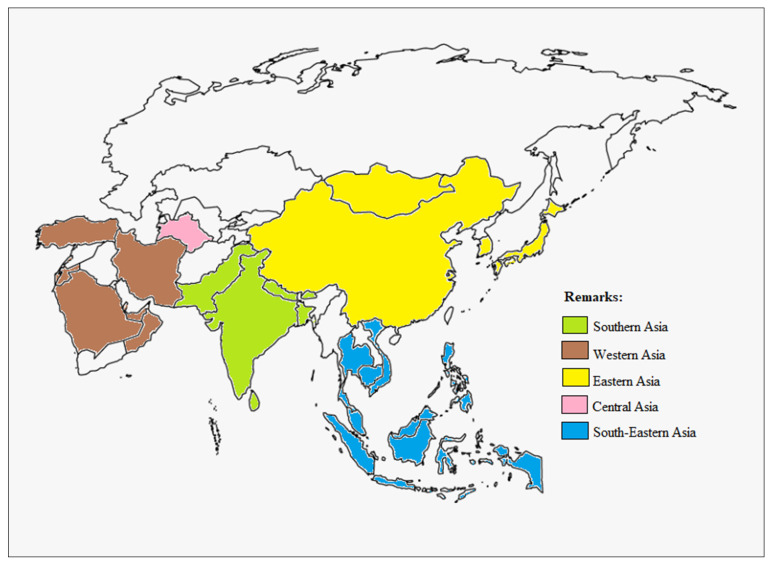
Thirty-two Asian countries that have implemented plastic pavement technology.

The idea of a plastic road was first developed by Dr. Rajagopalan Vasudevan (known as India’s Plastic Man) back in 2002 when he discovered that plastic has excellent binding properties similar to bitumen polymers [66]. The practice of using plastic waste as asphalt binders and mixtures is gaining more attention from road authorities due to the economic and environmental benefits [67]. In Asia, many countries have included plastic pavement technology as part of their road construction plan. Some countries also evaluated the actual performance of plastic-modified asphalt mixtures by constructing plastic road pavement in their countries (Figure 3).

Waste plastics are used in plastic roads by incorporating them into the asphalt mixture, either as an additive or modifier. In addition, the incorporation of waste plastics into the asphalt mixture can be carried out either by a dry mix method or wet mix method. The example of a typical process to incorporate plastic waste into asphalt mixture using the wet mix method involves the following steps:

1. Collection and Sorting: Waste plastics, such as plastic bags, bottles, and packaging materials, are collected from various sources, including recycling centers, waste management facilities, and consumer collections. These plastics may undergo sorting to separate different types and remove any contaminants.
2. Shredding and Granulation: The collected plastics are then shredded into smaller pieces or granulated into small pellets. This process helps reduce the plastic waste into a manageable size for incorporation into the asphalt mixture.
3. Mixing with Asphalt: The shredded or granulated plastic pieces are mixed with hot bitumen, which is the binder material used in asphalt. The heat softens the plastic and allows it to blend homogeneously with the bitumen. The plastic content in the mixture can vary depending on the specific requirements and desired performance of the plastic-modified asphalt.
4. Mixing with Aggregates: The plastic-modified bitumen is further mixed with aggregates, such as crushed stone or gravel, to create the final asphalt mixture. This mixture combines the properties of traditional asphalt with the benefits provided by the incorporation of plastic waste.
5. Paving and Compaction: The plastic-modified asphalt mixture is then transported to the construction site and laid down on the prepared road surface using conventional paving equipment. The mixture is spread and compacted to achieve the desired thickness and smoothness.
6. Road Application: After compaction, the plastic-modified asphalt forms a durable and flexible road surface, similar to conventional asphalt roads. The plastic content in the mixture enhances the strength, stability, and resistance to cracking, thereby improving the overall performance of the road.

It is important to note that the specific process and techniques for incorporating waste plastics into plastic roads may vary depending on the region, available technology, and desired outcomes. Ongoing research and development efforts aim to optimize the incorporation of waste plastics into asphalt mixtures and improve the overall sustainability and performance of plastic roads.

(a)  Southern Asia
  - Bangladesh

The Road and Highways Department of Bangladesh planned to build a 100 km nanotechnology acrylic road within one month. The results from a ten-month pilot project showed that the soils in Bangladesh were exceptionally suitable to be mixed with the acrylic polymer. The technology successfully reduced construction costs by at least 30%, with almost 70% of the building materials available in the country [68].

   ii.  Bhutan

In October 2015, Bhutan’s plastic road entrepreneur, Rikesh Gurung, built a 150-m pilot road in the capital Thimphu using bitumen mixed with plastic bottles and other plastic waste. The Green Road project was strongly supported by the Department of Roads, a municipal corporation of Bhutan, and financially supported by Bhutan’s Business Opportunity and Information Centre. The country expected to reduce the amount of bitumen imported from India by 40% and cut the amount of plastic waste going into landfills by 30–40% [69]. So far, 200 km of roads have been blacktopped using plastic [70].

   iii.  India

Since 2015, the government of India made it mandatory for all road developers to use plastic waste for road construction [71]. So far, eleven states have built almost 100,000 km of plastic roads that saved approximately $426.90 to $548.88 for every km of 3.75 m width of roads [72].

   iv.  Iran

Based on a literature search, only one recent paper related to plastic road marking was published. The researchers optimized the skid resistance and dirt pick-up properties of methyl methacrylate-based cold plastic pavement marking paints [73].

   v.  Nepal

A private company, Green Road Waste Management, conducted a 100-m pilot study road in Pokhara-14. The study used 2475 L of traditional bitumen mixed with almost 16% plastic wrappers from noodles, biscuits, milk packets, and tobacco. According to the civil engineer, Rajiv Subedi, the project cost only $243.95 and saved $2439.46 per km of road [70].

   vi.  Pakistan

Pakistan’s first plastic roads pilot test under the “World Without Waste” program was completed in December 2021 at F-Park and Ataturk Avenue, Islamabad, using almost 8 tons of polyethylene terephthalate recycled plastics [74]. The mega project was officially signed on 21 September 2021, involving the partnership between Coca-Cola Pakistan-Afghanistan, Capital Development Authority, and National Incubation Center. Once adopted widely, the initiative is expected to save the government valuable taxpayer money for road maintenance costs [75].

   vii.  Sri Lanka

South Asia Gateway Terminals, the first international container shipping private company in Sri Lanka resurfaced the port terminal road using plastic-modified asphalt concrete. The asphalt mixture incorporated single-use plastic waste as a substitute for a percentage of bitumen fossil fuel which reduced greenhouse gases, lowered the resurfacing frequency, cost efficiency, and improved road safety [76].

(b)  Western Asia
  - Armenia

The European Union, the Ministry for Territorial Administration and Development of Armenia, and the Urban Foundation for Sustainable Development developed a three-step model separating solid household waste from plastic waste involving about 30 Armenian communities. Then, they planned to obtain six building materials consisting of 80% sand and 20% plastic waste. Melted plastic will provide extra viscosity for the sand, which was generally provided by cement [77].

   ii.  Azerbaijan

MacRebur^®,^ a Scottish company specializing in plastic road construction, officially signed a £1 million contract with the Azerbaijani government to upgrade the road infrastructure network in the country. It is planned to build a 1690 km two-lane road using more than 500 tons of non-recyclable plastic waste. The project opens good prospects to connect people from China and Europe, as Azerbaijan is located at the center of the Trans-Caspian East-West Trade and Transit Corridor [78]. Since being launched in 2016, MacRebur^®^ has paved hundreds of miles of roads, paths, driveways, and parking lots in several Asian countries, including Turkey, Saudi Arabia, United Arab Emirates, Bahrain, Kuwait, and Japan [79,80].

   iii.  Bahrain

Following the previous success of recycling demolition and construction waste and reusing it as base layers on roads, Bahrain planned to repurpose used tires and plastic waste to construct roads or asphalt enhancers [81]. The implementation is part of Bahrain’s initiatives towards sustainable waste management to reduce, recycle, and repurpose 45–50% of their waste [82].

   iv.  Israel

An Israeli start-up company UBQ created a radical upcycling technology that transforms unsorted garbage into thermoplastic pellets [83]. The outputs are later used to make new materials, including road materials, bricks, bins, and flowerpots [84]. The closely guarded patented conversion process produces no carbon dioxide or toxic by-products. It also claimed to use little energy and no water [85].

   v.  Jordan

A study was conducted to utilize plastic waste as an additive in road aggregate modification. The study was one of the initiative solutions for the problem of plastic municipal solid waste management in Jordan. The addition of 4% plastic waste to 7% asphalt showed a significant positive effect on the properties of the hot mix asphalt [86].

   vi.  Kuwait

Kuwait built the plastic road using MacRebur’s technology [80]. There were a limited number of studies conducted by Kuwaiti researchers on the potential of plastic-mixed asphalt [87].

   vii.  Lebanon

A Lebanese industrial and environmental engineer, Mr. Ziad Abi Chaker, repurposed plastic waste to create durable manhole drain covers. He used single-use plastics to replace stolen metal covers in the south of Lebanon and near the Beirut River. Metal theft is common due to the deterioration of the country’s economic situation [88].

   viii.  Oman

Plastic waste contributes up to 21% of the total mass of Oman landfills [89]. The concept of plastic pavements is practicable in the country, as shown by several studies [90]. However, up to now there has yet to be an official implementation of the green road technology in Oman [91].

   ix.  Qatar

Qatar is among the highest waste generators (1.8 kg per person/day) on a per capita basis in the world [92]. Hence, it is recommended to utilize secondary raw materials such as high- and low-density polyethylene to sustain the requirement for natural aggregates in the road and building construction industry [93]. As the innovation of plastic roads in Qatar is under development, several development projects, including cycle paths, local roads in gated communities, or within FIFA 2022 facilities, can apply the idea [94].

   x.  Saudi Arabia

King Abdullah University of Science and Technology cooperated with Dow Chemical Company and NAPCO for Green Roads initiative to convert plastic waste in the kingdom into sustainable bitumen using a conventional modifier [95]. The material used for road construction projects around the King Abdullah University campus is expected to withstand heavier traffic loads, high temperatures in the Middle East, and lower road maintenance costs [96].

   xi.  Turkey

After China banned plastic imports on 1 January 2018, Turkey became a main importer of global plastic waste, especially from European countries [97]. Recently, the country utilized plastic road technology from the MacRebur^®^ company to pave their pathways, in addition to several laboratory studies on the potential of plastic-modified bitumen by local researchers [79,98,99].

   xii.  United Arab Emirates (UAE)

An Emirati start-up, Trident Trackway, has recycled plastic waste to create heavy-duty portable flooring products for the construction of temporary roadways, staging areas, and storage pads [100]. The products are meant to create smooth paths in the remote areas of rocky terrain in the UAE [101].

(c)  Eastern Asia
  - China

Milk Bottle Road is a partnership project between Chinese dairy company, Shiny Meadow; East China University of Science and Technology; and Dow Chemical Company [102]. The project used more than 6000 used milk bottles, easily recyclable bottles yet negligible by recyclers due to the high rate of contamination from leftover milk [103].

   ii.  Japan

Kao Corporation developed NEWTLAC 5000, an asphalt modifier made from discarded polyethylene terephthalate (PET) materials through proprietary processing. The NEWTLAC 5000 showed durability performance five times better than conventional asphalt modifiers, thus ensuring less microplastic production [104]. The newly developed technology has been adopted by Iwata City in Japan [105].

   iii.  Mongolia

Three field trials of polymer-modified warm-mix asphalt pavement were developed on South Gobi Road (2012) and Ulaanbaatar City Road (2012 and 2013) by the Korea Institute of Construction Technology. Using Low Energy and Low Carbon-Dioxide Asphalt Pavement (LEADCAP), an additive of a wax-based composition, the trials showed enhancement in rutting and crack resistance [106].

   iv.  South Korea

A prototype of temporary pavement blocks from waste plastic films was tested at low-speed driving sections with frequent traffic of heavy vehicles in Seoul. Based on the mechanical characteristics, accelerated weathering test, road pavement vehicle stability, environmental assessment, and field tests of the prototype, it can be applied as construction material for pothole emergency repair and backfilling structures for underground buried pipes [107].

(d)  Central Asia
  - Turkmenistan

Recently, the construction of the Ashgabat–Turkmenabat highway has been in progress. The Director of the branch of Westport Trading Europe Limited, Dr. Allaberdy Ilyasov, mentioned that polymers, especially products of polypropylene, polyethylene, and rubber waste, play an important role in improving the quality of roads in Turkmenistan. The creation of bitumen modifiers to produce high-quality road is one of the solutions to preserve the ecology of the country [108].

(e)  South-Eastern Asia
  - Cambodia

The Ministry of Public Works and Transport of Cambodia is looking into a proposal proposed by IKEE Co Ltd., Osaka, Japan. As Cambodia receives massive rainfall, plastic-waste converted concrete is a good choice for waterproof and long-lasting roads [109].

   ii.  Indonesia

The government of Indonesia collaborated with several partners including TCE India and Chandra Asri Petrochemical to construct plastic-tar roads in a few areas such as Kudus, Central Java (39,000 m^2^), and BSD City, Banten (15,518 m^2^) [110,111].

   iii.  Malaysia

In 1997–1998, the Public Works Department of Malaysia built runways and taxiways for Kuala Lumpur International Airport using 350,000 tons of plastic-modified asphalt. The mixture contained synthetic polymer of low-density polyethylene, ethylene methacrylate, and styrene-butadiene-styrene for the pavement of Runway 1, Runway 2, and Taxiway, respectively. In September 2017, 175 m of plastic asphalt was laid for the first time on the public road of Temerloh, Malaysia. It showed better mechanical performance in terms of rutting and cracking resistance compared to conventional asphalt [112]. In addition, Setia Bintang Engineering Sdn Bhd, a construction and civil engineering company and exclusive distributor for MacRebur^®^ in Malaysia, Thailand, and Indonesia, planned to pave roads in Malaysia using Plastic Binder Extender technology [113].

   iv.  Philippines

A Philippines company, San Miguel Corporation, has laid down 1500 square meters of plastic road developed by Dow Chemical Co., using 900 kg of soft plastic scraps for its first project. The initial findings showed that the plastic road exceeded government standards and were stronger and more durable than those made with conventional asphalt [114].

   v.  Singapore

Magorium, a Singaporean start-up, ran a trial test by paving plastic roads on private-owned lands at a factory site in Tuas and a condominium in Marymount. According to Magorium’s chief executive officer, Ms. Oh Chu Xian, their recycling technology is capable of breaking down six types of discarded plastic into three different forms (powder, shreds, or pellets) before being added into bitumen mixture. In addition, the company is collaborating with the National Environment Agency and the National Water Agency of Singapore to conduct environmental risk assessments, particularly on the surface runoffs of microplastics [115].

   vi.  Thailand

As a strategy to maximize the value of plastic following the concept of circular economy, Chemicals Business, SCG has partnered up with Dow Thailand Group to construct recycled plastic roads. A prototype project of 220 m in length, 3 m in width, and 6 cm thick road was constructed in RIL Industrial Estate, Rayong. Next, 2600 m^2^ of plastic roads will be constructed in Amata City Industrial Estate, which will require about 1.3 tons of plastic waste, equivalent to 100,000 plastic bags [116].

   vii.  Timor-Leste

The Plastics Solutions Alliance, which was created in October 2019, addressed the staggering amount of plastic waste in Timor-Leste by collaborating with Heineken, Caltech, USAID, Mercy Corps, and the Korean International Cooperation Agency to create a value chain for reducing single-use plastic. Under the alliance, Caltech had set up a plastic waste recycling facility that turns the waste into road resurfacing materials. Whereas Heineken cooperated with the University of Timor-Leste by contributing shredded plastic for asphalt road research [117].

   viii.  Vietnam

In November 2019, 1.4 km of road was completed at a DEEP C industrial zone in Haiphong, Vietnam. The project was a collaboration between Dow Vietnam and DEEP C (a shareholder company between Belgian and Hai Phong People’s Committee). Two laboratory tests conducted by Vietnam Maritime University showed that the post-consumer recycled plastic pavement fulfilled the technical and safety requirements of the Vietnamese government [118].

Among 48 countries in the Asia region, 32 countries have invested in the research and development of recycled plastic road technology, whether in laboratory studies, field trials, or real implementation of plastic roads. Some countries including Bhutan, Israel, and Singapore managed to set up their own start-up companies based on state-of-the-art technology. Whereas other countries collaborated with private companies or non-governmental agencies to build the plastic roads. In general, most countries agree that plastic road pavement helps in reducing the plastic waste issue in their countries.

### 3.2. Difference between Polymer-Modified Asphalt and Plastic-Modified Asphalt

Polymer-modified asphalt and plastic-modified asphalt are two distinct types of asphalt mixtures that incorporate different materials to enhance their properties. Here is a breakdown of the differences between the two:

(a)  Polymer-modified asphalt: Polymer-modified asphalt refers to asphalt mixtures where polymers, such as styrene-butadiene-styrene (SBS) or styrene-butadiene rubber (SBR), are added to the asphalt binder. These polymers are usually derived from synthetic rubber or thermoplastic materials. The polymer content in polymer-modified asphalt is typically higher than that of plastic-modified asphalt.
  1. Purpose: The addition of polymers is aimed at improving the performance characteristics of the asphalt. Polymers enhance elasticity, flexibility, and resistance to deformation and cracking, making the asphalt binder more durable and able to withstand heavy traffic loads and harsh weather conditions.
  2. Properties: Polymer-modified asphalt exhibits improved rutting resistance, reduced cracking, and increased elasticity compared to conventional asphalt. It also offers enhanced adhesion to aggregates and improved resistance to moisture damage.
  3. Application: Polymer-modified asphalt is commonly used in high-stress areas such as intersections, heavy traffic zones, and airports. It is also employed in regions with extreme climates where asphalt durability is crucial.
(b)  Plastic-modified asphalt: Plastic-modified asphalt, as discussed earlier, involves incorporating waste plastics, such as plastic bags, bottles, and packaging materials, into the asphalt mixture.
  1. Purpose: The primary objective of adding plastic waste to the asphalt is waste management and recycling. By incorporating plastics into roads, it offers a sustainable solution for plastic waste disposal, reducing landfill usage and environmental pollution.
  2. Properties: Plastic-modified asphalt exhibits improved resistance to cracking, better durability, and reduced moisture susceptibility compared to conventional asphalt. The plastic content helps enhance the strength and stability of the road surface.
  3. Application: Plastic-modified asphalt is suitable for various road applications, including highways, urban roads, and residential streets. It is considered a greener alternative to conventional asphalt and is particularly relevant in regions with significant plastic waste generation.

In summary, the main distinction lies in the materials added to the asphalt mixture. Polymer-modified asphalt incorporates synthetic rubber or thermoplastic polymers to enhance performance, while plastic-modified asphalt uses waste plastics for waste management purposes, improving road properties along the way. Both approaches contribute to the development of more resilient and sustainable road infrastructure.

## 4. Considering Plastic Roads in Asian Countries

In line with the population and economic growth in the Asian region, there are yearly increasing demands for efficient transport systems, particularly on the land road infrastructure [119]. Most of the conventional bitumen grades provide satisfactory adhesion and mechanical performances as asphalt pavement for most traffic and climatic conditions [120]. Nevertheless, the performances decline due to several reasons including an increase in axle loads, heavy traffic, and severe climate conditions; thus, leading to earlier road failures that require high-cost maintenance [121].

### 4.1. Plastic-Modified Asphalt

One of the promising solutions to improve pavement performance at a reasonable cost is using polymer-modified bitumen. The modification technology depends on the specific applications and techniques, including surface dressings, thin surface course systems, porous asphalt, surface courses for heavily trafficked roads, bases for heavily trafficked roads, anti-cracking membranes, or bridge waterproofing layers [122]. Thermoplastic elastomers (such as styrene-butadiene, styrene-butadiene-styrene, polyisobutene, polybutadiene, and polyisoprene) and thermoplastic polymers (such as ethylene-vinyl acetate, ethylene-methyl acrylate, polyethylene, polypropylene, and polystyrene) are two types of bitumen modifier that have been experimented with for many years by researchers and chemists in the road construction industry, while complying with product standards in different countries [123,124].

Many previous studies reported better mechanical properties of plastic-modified asphalt including Marshall stability, indirect tensile strength, indirect tensile strength ratio, and resistance to permanent deformation and fatigue [125]. Consequently, it produced a roadway with better water resistance, better load resistance, less bitumen usage, fewer potholes, and a longer lifecycle [72]. However, plastic pavements require more points to consider beyond engineering capability. From the perspective of Sustainable Development Goals (SDGs) that are progressively adopted as a foundation for both private and state sector stakeholders promoting sustainability practices, plastic waste links to several of the SDGs including SDG 11 (Sustainable Cities and Communities) through waste management; SDG 12 (Responsible Consumption and Production) through plastic consumption in the circular economy; and SDG 13 (Climate Action) as plastic derives from fossil fuels [126].

### 4.2. Consideration Aspects

There are several aspects that need to be considered before constructing plastic roads in Asian countries, including:

- Governmental policies

Until 2021, the total nominal gross domestic product for Asian countries reached $36.8 trillion, with China in the top rank. However, the economic development level for most of the countries in Asia was relatively low [127]. As a strong economic structure is urgently in need, low- and moderate-income countries have to overcome economic issues to enable sustainable development in their countries [128]. For example, the combination of housing policy and rapid urbanization in Xiamen Island, China, has increased the efficiency of transport energy consumption for the residents. There was a transition that occurred from older urban village settlements to newer commercial developments by emphasizing the public transport development and enhancement of integrated road construction and planning [129].

b.  Stakeholders’ perspectives

When plastic roads are marketed as a win–win solution, municipalities assume that they are performing a positive effort for the environment as well as improving the economy of the local community, as in the case of Gurugram, India [130]. Nonetheless, many environmentalists expressed their concerns regarding the leaching and fuming of harmful chemicals from plastics, the high hidden external costs of plastic road maintenance, and the regression of plastic-free society force [131].

c.  Geographical characteristics

In general, Asian countries have different climates, topography, soil, hydrology, vegetation cover, and terrestrial ecosystems that shape their geographical characteristics [132]. With various climate zones (i.e., tropical, arid, temperate, and cold climates), complex terrain (including majestic mountains, vast plateaus, and fertile plains), more than 30 types of soil (such as leptosols, gleysols, podzols, and cambisols), different runoff depth and precipitation rates (with the highest runoff depth is in the South and Southeast Asia regions and the lowest is located in Northwest China and some Arabian Peninsulas), and heterogenous vegetations (including forests, grasses, shrubs, and croplands), the Asian continent is prone to the damaging impacts of climate change that leads to devastating natural disasters including heat waves, drought, and flood events [132,133].

d.  Supply of raw materials and manpower

It is estimated that 7000 cubic meters of aggregates are needed to construct a one-kilometer length of bituminous highway pavement, with an additional 300 cubic meters for annual maintenance [134]. However, not all areas in a country have good quality aggregates and gravel. As an example, in Maraimalai Nagar, India, the idea of a plastic road was adopted to solve the raw materials scarcity and plastic waste problem [135]. Ironically, after a year, the project was abandoned since they failed to collect enough plastic for the road construction, despite offering reasonable wages of 4-gram gold coin in exchange for 500 kg of single-use plastics to the residents [131].

## 5. Environmental and Human Health Adverse Effects of Plastic Roads

**Figure 4:**
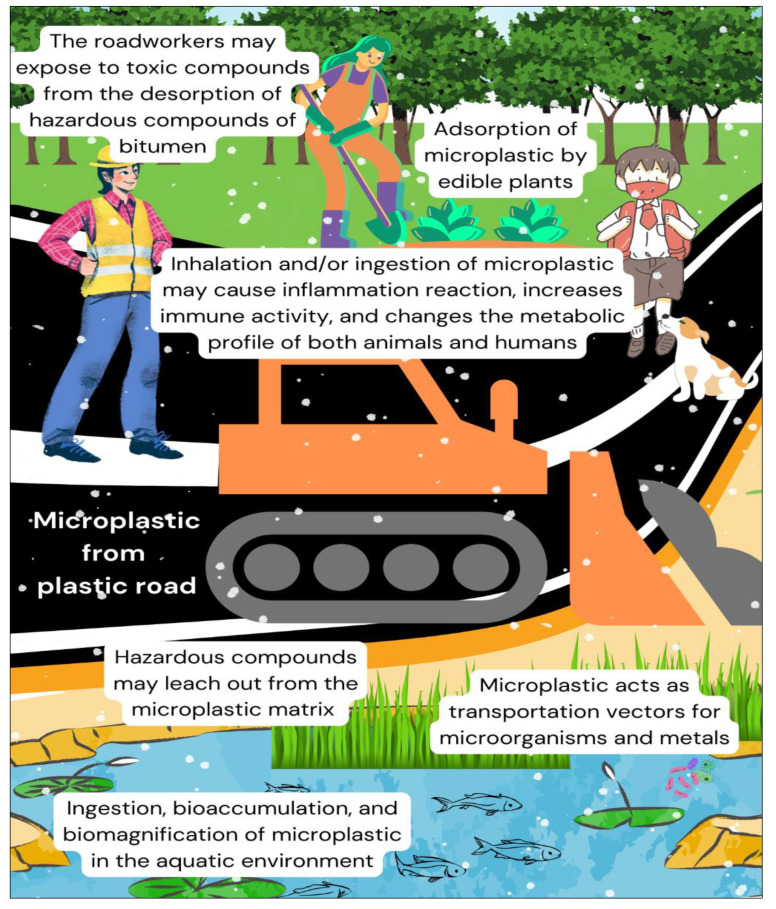
Infographic on adverse effects of plastic roads on the environment and human health.

Currently, there are two adverse issues related to plastic roads that receive less emphasis in the previous studies, namely environmental and human health aspects [136]. Based on Pawar et al. (2021) [136], the environmental and human health adverse effects of plastic roads are explained as an infographic in Figure 4 below.

One of the main concerns of plastic roads is the breakdown of microplastics into the environment [137]. Despite being highly resistant to biodegradation, extensively used plastic polymers for road pavement such as polypropylene and polyethylene can be degraded by heat, oxidation, light, ionic radiation, hydrolysis, mechanical shear, and pollutants such as carbon monoxide, sulphur dioxide, nitrogen oxide, and ozone [138,139,140]. In addition, several studies showed poor chemical compatibility between plastic polymers and bitumen, leading to phase separation issues of the components, especially for polymers with high molecular weight and low maltene fractions [141,142,143]. Polymer phase separation is an undesirable condition that greatly affects the mechanical performance of asphalt at the microscale and might clog the tubes and nozzles of the pavement pump [144,145]. Moreover, phase separation may cause leaching out of the recycled plastic from asphalt mixtures, consequently polluting the environment [146].

### 5.1. Adverse Effects on the Environment and Human Health

For over a decade, microplastic pollution has become an emerging global concern, mainly in marine ecosystems [147]. Recently, the pollution of rivers, soil, and atmosphere by microplastics has gained more attention [148,149,150]. It is estimated that every year between 1.15 and 2.41 million tons of plastic waste enter the marine waters originating from rivers, mostly from the Asian rivers [151]. On the other hand, the atmospheric microplastics are horizontally transported by wind up to 95 km distance, and finally deposited in the water and soil environments [152,153,154].

In a polluted environment (mostly in the aquatic), microplastic debris is transferred to the higher-trophic-level organisms via ingestion, bioaccumulation, and biomagnification mechanisms [155]. Subsequently, it causes inflammation reactions, increases immune activity, and changes the metabolic profile of both animals and humans [156,157]. In addition, edible plants such as vegetables can absorb microplastic particles into the roots and pose harm when consumed directly by humans [154].

In addition to particle toxicity, microplastics could present chemical and biological risks to organisms in the environment [158]. Monomer and chemical additives may leach out from the microplastic matrix, producing hazardous compounds including plasticizers, flame retardants, stabilizers, and biocides [159]. Several plastic additives such as polybrominated diphenyl ethers and hexabromocyclododecane have been banned in European and North American markets due to their persistence, potential for bioaccumulation, and risks of toxication [160]. Whereas common plastic additives such as formaldehyde and bisphenol A are classified as carcinogen category 1B and hormone disruptor, respectively [161].

In addition, post-consumer recycled plastics normally used for plastic road construction contain hazardous non-intentionally added substances with various concentrations and nature depending on their composition, manufacturing, and recycling process [162,163]. These substances are concerning since the high temperatures (up to 180 °C) used during the production of bitumen can generate the desorption of the hazardous compounds and expose roadworkers to toxic compounds [164].

Furthermore, the high surface area of microplastics makes them favorable transportation vectors for microorganisms (such as *Vibrio* spp. and *Escherichia coli*) and chemicals (including heavy metals such as zinc, plumbum, and cadmium and persistent organic pollutants such as polyaromatic hydrocarbons, organochlorine pesticides, and polychlorinated biphenyls) [165,166]. The ingestion of these pathogen- and toxicant-loaded microplastic particles could lead to infections and toxicity in biotic organisms [167,168]. Thus, studying microplastic contaminants is vital to maintain a healthy environment for humans, animals, and plants.

### 5.2. Limited Risk Assessments

To date, no longitudinal study has been conducted by road construction industries or researchers on the mechanism of plastic abrasion from plastic-modified bitumen and its impact on the ecosystem [126]. A recent report by the Scottish Road Research Board proposed that waste-derived plastic should be stopped from being used in the road surfacing, bound, and unbound layers of road construction, unless in trial situations [169]. The oldest plastic roads being built are less than eight years old with a life expectancy of up to three times more than the traditional asphalt pavement, thus resulting in unclear long-term performance and environmental and health risks of plastic-modified asphalt [94].

Many standard tests empirically test the asphalt performance temporarily under controlled laboratory conditions that do not represent the real scenarios in the field [170]. Alternatively, accelerated pavement testing using Heavy Vehicle Simulator or Pavement Fatigue Carousel is currently being used to test the surface and structural changes of asphalt [171,172]. However, some parameters including gaseous emissions level when laying hot asphalt and the release of microplastics when plastic-modified roads are open to traffic due to abrasion exerted by vehicle tires cannot be assessed via accelerated pavement testing [164].

Other crucial yet limited studies had been conducted on the environmental impact of plastic roads via its life cycle assessment (LCA) in which most of the studies depend on secondary data and modeling rather than primary data collected directly from the plants [173]. There are also no LCA data on the release of microplastic particles from plastic-modified roads upon traffic and road wear [164]. The phenomenon of microplastic release from roads is debatable as its position in the asphalt pavement depends on several factors including the ways plastics are incorporated into the bitumen (wet or dry method), the type of plastics used (recycled or virgin plastics), as well as external climatic conditions [164].

Last but not least, as plastic road preparation requires higher temperatures than the melting point of plastic, and the incorporation of plastic particles generates hazardous fumes from both bitumen and plastics [174]. Until today, there are limited data on the real-time toxicological assessment of the total amount of fumes emitted from plastic-modified asphalt among roadworkers [175,176].

## 6. Conclusions

Plastic roading is one of the good initiatives for reusing plastic waste, especially in Asian countries with a high population and many plastic manufacturers and consumers. Its better performance than conventional asphalt roads gives it a bright potential for the plastic road idea to be expanded on a large scale in Asia. However, as the technology is considered relatively new, and without standard analysis on its environmental and health risk assessment, the implementation of plastic roads requires strong collaboration between the government, private sectors, and academics to gather holistic information to fully understand the impact of paving plastic-modified asphalt on living organisms and ecosystems.

Implementing plastic roads is indeed a positive initiative for reusing plastic waste, particularly in densely populated Asian countries with a significant plastic manufacturing and consumption rate. The superior performance of plastic-modified asphalt compared to conventional asphalt roads adds to its potential as a viable solution. This presents an opportunity for the widespread adoption of plastic roads on a large scale in Asia. However, it is important to acknowledge that plastic road technology is relatively new, and comprehensive analysis regarding its environmental and health risk assessment is still lacking. Strong collaboration between government entities, private sectors, and academic institutions is essential to ensure the successful implementation of plastic roads. This collaborative effort will help gather holistic information and conduct thorough studies to fully understand the impact of paving plastic-modified asphalt on living organisms and ecosystems. By conducting comprehensive environmental and health risk assessments, addressing concerns related to potential pollution, leaching of toxic substances, and long-term effects on soil, water, and human health will be possible. This information will enable policymakers, industry stakeholders, and researchers to develop appropriate guidelines and regulations for the safe and sustainable implementation of plastic roads. The involvement of government bodies is crucial to provide regulatory frameworks and standards, while private sectors can contribute by investing in research and development, as well as the establishment of proper manufacturing processes. Academic institutions can play a vital role in conducting scientific studies, monitoring the impacts of plastic roads, and providing unbiased evaluations. This collaborative approach provides valuable insights, leading to a comprehensive understanding of the benefits, drawbacks, and potential risks associated with plastic-modified asphalt. This knowledge will contribute to developing informed decisions and strategies to maximize the positive impact of plastic roads while minimizing any potential adverse effects on the environment and human health.

Prospects of Plastic Roads:

1. Waste management: Plastic roads provide a sustainable solution for managing plastic waste by repurposing it into road construction materials. This helps reduce plastic pollution and landfill usage.
2. Improved road performance: Plastic-modified asphalt can enhance the durability, flexibility, and resistance to cracking and deformation of road surfaces. This can result in longer lasting and more resilient roads, reducing the need for frequent repairs and maintenance.
3. Cost savings: Plastic roads have the potential to offer cost savings over time due to their improved longevity and reduced maintenance requirements. This can be particularly beneficial in areas with limited resources for road infrastructure.
4. Environmental benefits: By incorporating waste plastics into roads, there is a reduction in the demand for virgin materials such as bitumen, derived from fossil fuels. This can contribute to conserving natural resources and reducing carbon emissions associated with the production and transportation of conventional road construction materials.

## 7. Recommendation and Contribution of the Study

Several significant factors must be properly considered and handled when considering plastic roads. These factors consist of:

1. Environmental impacts: Although plastic roads may help reduce plastic waste and the need for virgin materials, it is important to consider their overall environmental impact. This includes assessing the emissions produced during the manufacturing process, the potential environmental impact of toxic chemicals seeping from plastic components, and the long-term consequences on soil and water quality.
2. Impacts on human health: The use of plastic in road building raises questions regarding potential hazards to human health, particularly if harmful materials from plastic seep into the environment. To protect the health of those involved in the manufacture and installation of plastic roadways, as well as the local communities, thorough health risk evaluations should be carried out.
3. Control of quality: It is essential to maintain the plastic-modified asphalt’s reliability and performance. To guarantee that the plastic components fulfill necessary standards and operate as expected under various weather conditions and traffic loads, it is crucial to create standardized production procedures and quality control measures.
4. Limitations of recycling: Although plastic roads offer a way to make use of discarded plastic, it is vital to take them into account. Some plastics could be harder to recycle or might only have a few recycling choices. It is essential to create effective recycling methods and investigate other uses for plastic trash than making roads.
5. Performance over time: Plastic-modified asphalt must show endurance and longevity. To determine whether plastic roads can resist the anticipated lifespan of conventional asphalt roads, it is required to assess their long-term performance under various climatic conditions, traffic loads, and maintenance schedules.
6. Economic viability: It is crucial to evaluate the economic viability of plastic roadways. This includes comparing the cost-effectiveness of generating plastic-modified asphalt to conventional asphalt while taking into account elements such as material accessibility, production prices, and upkeep needs.
7. Policy and regulatory framework: It is essential to create proper policies, rules, and standards before implementing plastic roads. To ensure compliance with environmental and health legislation, this includes establishing standards for material selection, quality control, and monitoring practices.

Future Work:

1. Comprehensive research: Further research is necessary to gain a deeper understanding of plastic roads’ environmental and health impacts. This includes studying the leaching behavior of plastic components, assessing the potential effects on soil and water quality, and conducting lifecycle assessments.
2. Standardization and guidelines: Developing standardized protocols, material specifications, and guidelines for plastic road implementation can ensure consistency and safety. This will require collaboration between stakeholders, including government bodies, researchers, and industry experts.
3. Technological advancements: Continued technological advancements can help optimize the manufacturing process of plastic-modified asphalt, improve recycling techniques, and enhance plastic roads’ overall performance and sustainability.
4. Knowledge sharing and collaboration: Encouraging knowledge sharing and collaboration between different regions and countries can facilitate the exchange of best practices, lessons learned, and research findings related to plastic road technology. This can help overcome challenges and promote the widespread adoption of plastic roads.

Overall, while plastic roads offer promising prospects in waste management and road construction, addressing the associated challenges and limitations through research, standardization, and continuous improvement will be crucial for their successful implementation and long-term viability. By carefully considering these variables, it is possible to address the opportunities and problems related to plastic roads and encourage their effective implementation as a long-term method of handling plastic trash in road building.

## Figures and Tables

**Table 1:** Average amount of waste plastic generation in Asian countries [14].

Country	Total Plastic Waste (Mt)		
	2016	2017	2018
China	49.04	49.19	49.71
India	17.48	17.58	17.66
Japan	10.95	11.07	11.19
Turkey	6.13	6.21	6.28
Thailand	5.85	5.88	5.96
Pakistan	5.30	5.40	5.51
South Korea	4.40	4.39	4.38
Vietnam	3.28	3.29	3.30
Iran	3.20	3.19	3.24
Saudi Arabia	2.97	3.05	3.11
Indonesia	2.74	2.95	3.01
Philippine	2.53	2.58	2.61
Malaysia	2.45	2.60	2.65
Singapore	1.19	1.22	1.24
Israel	1.02	1.02	1.03
Yemen	0.91	0.92	0.93
Sri Lanka	0.54	0.55	0.56
Mongolia	0.46	0.46	0.46
Tajikistan	0.35	0.36	0.36
Qatar	0.24	0.24	0.25
Turkmenistan	0.16	0.16	0.16
Brunei	0.05	0.05	0.05

## Data Availability

All the data have been included in this paper.
